# 

**Published:** 1915-11

**Authors:** 


### ANNOUNCEMENTS

### REMOVAL NOTICE.

Dr. B. Holly Smith and Son have removed their office to 405 North Charles Street, Baltimore.

---

### OFFICERS OF NATIONAL DENTAL ASSOCIATION.

At the recent meeting of the N. D. A, held in San Francisco the following officers were elected for the ensuing year:

President, Dr. T. P. Hinman, Atlanta, Ga.

First Vice President, Dr. H. B. Tileson, Louisville, Ky.

Second Vice President, Dr. A. M. Flood, San Francisco, Cal.

Third Vice President, Dr. W. A. Giffen, Detroit, Mich.

Secretary, Dr. Otto U. King, Huntington, Ind.

Treasurer, Dr. A. R. Melendy, Knoxville, Tenn.

Louisville, Ky., was selected as the place for holding the 1916 meeting of the association.

---

### NATIONAL ASSOCIATION OF DENTAL FACULTIES.

The National Association of Dental Faculties will meet at the Hotel Radisson, Minneapolis, Minn., Friday and Saturday, January 28, 29, 1916.

C. C. Allen, *Secretary,*
N. W. Cor. 10th and Troost, Kansas City, Mo.

---

### PANAMA-PACIFIC DENTAL CONGRESS.

The Panama-Pacific Dental Congress has passed into history, and in accordance with the opinion very generally expressed by those in attendance, the meeting was a decided sucess.

The Pacific Dental Congress Commission of 1915, the corporation now in charge of all matters relating to the Congress and the publication of the transactions, desires to announce that a copy of the complete transactions, when published, and a copy of the official souvenir program, will be sent to any one making application therefor to the Secretary of the Commission, Dr. Arthur M. Flood, 240 Stockton Street, San Francisco, Cal., and forwarding the fee of ten dollars.

Those subscribing for these extra copies of the transactions can not be regarded as being members of the Congress, not having applied for membership before the meeting, or being in attendance at the same, but we believe these transactions will be a very valuable addition to the history of dentistry, and the souvenir program, containing at it does many items of historical interest and value, will be acceptable to any member of the profession.

PACIFIC DENTAL CONGRESS COMMISSION OF 1915.

Arthur M. Flood, D.D.S., Secretary.

---

### REPORT OF ACTION TAKEN AT A MEETING OF THE NATIONAL MOUTH HYGIENE ASSOCIATION, MAY 29, 1915.

The following report summarizes for your attention important action taken at the meeting of the Association held in the city of Washington on May 29th of this year. Plans for carrying out such action are now matured and ready to be put into operation as described in the pages following.

Full details regarding the history and accomplishments of the Association to date, together with the information contained in this report will be published in early issues of leading dental journals.

At the meeting, held in the city of Washington, D. C., several important matters were presented and passed upon.

The work of the officers was recognized by their re-election.

The constitution and by-laws were amended to conform to the laws of the District of Columbia, and facilitate the completion of the incorporation of The National Mouth Hygiene Association as a corporation not for profit.

The principal action necessary to conform to law in this connection was the creation of a Board of Trustees. The thirteen members of the existing Board of Governors, including the Secretary-Treasurer, were appointed to this Board of Trustees, and two additional members, the President of the Association and the Chairman of the Oral Hygiene Committee of the National Dental Association, making the total number fifteen.

It was also necessary to change the formal title of the Secretary-Treasurer to "Secretary-Treasurer General."

Further changes in the constitution were made in order to enable the Association to carry out its purposes and policies; and a preamble to the constitution was adopted setting forth these purposes and policies as follows:

1. The teaching of Mouth Hygience, and its relation to better health, increased mental and physical working efficiency, and consequent greater happiness;

2. To provide both the expert service and the funds necessary to enable the organized dental profession in every community to do those things that are for the best interests of its people;

3. To direct the attention of parents and guardians to the importance of dental service, especially in childhood;

4. To eliminate the dental fakirs, charlatans and fraudulent advertisers who subsist on the ignorance and credulity of the public;

5. To teach Preventive Dentistry and to recommend the employment of the highest type of professional services;

6. To promote the efficiency of the organized dental profession individually and collectively and to give it a wider, more responsive and more intelligent field in which to work;

7. To bring together actively not only the serious workers in the dental profession, but people of all other professions and vocations, and to enlist their united interest and co-operation in the expansion of the propaganda of Preventive Dentistry and Mouth Hygiene;

8. To act as a servant, aid and auxiliary, to both the organized dental profession and the American public to secure and retain the highest and most permanent benefits for all, through the realization of the objects first stated.

The general financial problems of the Association also came up for consideration and important action taken which is expected in time to result in larger and more dependable sources of revenue for the local and national philanthropic work.

Annual dues of active members were raised from $1.00 to $2.00 and new methods adopted for the sale and distribution of Mogene Dental Products.

Members are, of course, familiar with the fact that Mogene Dental Cream has for some time been manufactured and distributed under supervision and guaranty of the National Mouth Hygiene Association with a binding guaranty that that portion of the proceeds of sale received by the Association shall be devoted to the national and local philanthropic work in the cause of Mouth Hygiene.

The Association was led to this action for two reasons: First, because it realized that there was a logical and effective means of increasing the funds available for prosecution of its philanthropic propaganda. Second, because it felt the need of a dentifrice which it could conscientiously recommend to the general public and the dental profession as one made under the supervision and guarantee of the National Mouth Hygiene Association.

Leading dentists throughout the country had requested the Association to do this for the protection of the general public, who are unable to distinguish between reputable preparations and those containing ordinary commercial chalk, injurious disinfectants and antiseptics or other unsatisfactory ingredients.

The Association did not undertake the actual distribution of Mogene, however, until careful study had been given to the subject, and investigation showed that it was feasible to produce a dentifrice of the requisite quality, and to market it successfully.

It is eminently just and fitting that the Association, which has so much encouraged the use of dentifrices, should derive some support from their sale, instead of having its philanthropic efforts accrue wholly to the private profit of established manufacturers of dental preparations. Nothing undertaken by the Association, however, will be antagonistic to the interests of the manufacturers of *reputable* dental preparations—on the contrary, the success of The National Mouth Hygiene movement is greatly to their advantage.

Commercial distribution, of course, implies practical business methods, and the plans adopted at the meeting on May 29th will, it is felt, put the distribution of Mogene on a sound and thoroughly satisfactory basis.

To this end The National Mouth Hygiene Association authorized its officers to form an organization to be known as The Mogene Laboratories Company, which will manufacture Mogene Dental Cream and also Mogene Tooth Powder under supervision and guaranty of the Association, and distribute them through regular commercial channels by the most efficient methods of modern merchandising.

In addition, the plan for the distribution of Mogene products provides as heretofore for co-operative memberships in The National Mouth Hygiene Association or its auxiliaries, available on payment of one dollar ($1.00) by any person interested in promotion of the work. In return he receives four full packages of Mogene Dental Cream, which is the regular amount delivered at retail for one dollar.

This Co-operative Package has already shown great earning possibilities to the direct benefit of the local auxiliaries, and should develop tremendously with full realization of the opportunity by all active members.

The Co-operative Package, being distributed at the full retail price through the auxiliaries, can be sold at less "overhead" expense and approximately 50 cents out of every dollar can be applied to the funds available for local work.

It also is of great assistance in soliciting a large Co-operative Membership—thus enlisting more and more persons in the cause of Mouth Hygiene—because the recipient gets back the full value of his membership fee in a dentifrice of exceptional merit, and knows that he is at the same time contributing half the purchase price to philanthropic work.

Another extremely important advantage of this package is that it helps the sale of Mogene through the regular commercial channels from which further returns are made available for local and national work. Those who are thus once introduced to Mogene almost invariably become enthusiastic users because of its pleasant flavor, entire freedom from grit or other injurious ingredients, and its superior cleansing properties.

The sale of Mogene to the great general public will be prosecuted by the most efficient methods of modern merchandising. Especially in cities where the local work of the Association is well established.

Mogene Dental Cream and Tooth Powder will be advertised to the public, and placed on sale at reputable drug stores and department stores. Each case of one dozen packages will contain a *participation certificate* to be held by the dealer until collected by the authorized local representative of The National Mouth Hygiene Association or the local dental organization. Return of these certificates to the Executive Offices of The National Mouth Hygiene Association will entitle the local auxiliaries to pro rata participation in such funds as are to be set aside for the purpose from the general revenue of the Association.

This participation plan has been carefully worked out and should prove to be better than the benefit checks formerly used.

In all matters relating to receipt and disbursal of funds, whether from contributions, sale of Mogene products or any other source, the Association has made it impossible for any of its present or future officers to apply its revenue to any save its recognized philanthropic needs.

It is not only bound by its incorporation as an association not for profit, but by a definite guaranty—which has been widely published and is printed on the containers of Mogene products—as well as by contracts with auxiliaries and other local organizations.

To make doubly sure, the Association has appointed a National Board of Censors composed of the following well-known editors of representative dental and educational journals and the public press:

Dr. C. N. Johnson, Chicago, Ill. Editor of *The Dental Review.*

A. E. Winship, Boston, Mass. Editor of *The Journal of Education.*

Dr. Geo. W. Clapp, New York City. Editor *The Dental Digest.*

Wm. C. Bruce, Milwaukee, Wis. Editor *American School Board Journal.*

Henry G. Williams, Athens, Ohio. Editor *The Ohio Teacher.*

Dr. L. P. Bethel, Columbus, Ohio. Editor *The Dental Summary.*

Frank B. Noyes, Washington, D. C. President Associated Press.

These gentlemen are empowered to examine the books and records of The National Mouth Hygiene Association and its subsidiary organizations. Should they discover any evidence that the Association is not carrying out its obligations to the general public, and to the dental profession, it is their duty to give such evidence the widest publicity in the journals which they represent.

Considering the matters discussed and action taken, the meeting of May 29th will go on record as one of the most important in the history of The National Mouth Hygiene Association.

Cleveland, Ohio, October 26, 1915.

---

### FORSYTHE POST GRADUATE WORK.

By the opening of a post graduate school of orthodontia, the Forsythe Dental Infirmary for children is fulfilling one more of its functions as conceived in its original plans, and one which was characterized by President Emeritus, Charles W. Eliot, in his address at the dedication exercises, as one of its most important, influential duties, the education of the profession and the public.

While the school is not the first to be established in this important field of dentistry, it is the first to adopt a full academic year of instruction. The curriculum is comprehensive and includes not only the technical dental subjects, but also all of the allied medical branches that have a bearing on the development of the child. The unsurpassed clinical facilities of the institution, which are already demonstrated, will provide the student ample opportunity to acquire the practical experience necessary. Emphasis will be laid on preventive orthodontia. The faculty includes not only local men prominent in their branches, but also a large number of specialists from the East and Middle West.

---

### PROGRAM OHIO STATE DENTAL SOCIETY.

The Ohio State Dental Society Semi-Centennial Meeting and Dedication of the American Miller Memorial at Columbus, December 7, 8, 9 and 10, 1915.

The program of papers, so far as completed, comprises:

Dr. E. C. Mills, President's Address.

Dr. Herman Prinz, "On Causes Concerning Susceptibility and Immunity to Dental Caries."

Dr. Geo. H. Wilson, "Some Problems in Mounting Full Artificial Dentures."

Dr. Chas. C. Voelker, "The Place of Silicate Cements in Dentistry."

Dr. Geo. E. Johnson, "How to Read X-Ray Films."

Dr. J. H. J. Upham, "Pyorrhoea Alveolaris from a Medical Viewpoint."

Fifteen-minute papers on practical subjects by:

Dr. W. O. Hulick, "Are Crowns and Bridges a Menace to Health?"

Dr. J. P. Henahan, Conductive Anesthesia in the General Practice of Dentistry."

Dr. C. K. Teter, Management of Difficult Extractions."

Dr. H. V. Cottrell, "Accessories to Articulation."

Dr. Gillett Hayden, "Differentiation Between Average Tooth Cleaning and Prophylaxis."

Dr. L. E. Custer, Subject to be announced.

Explanation of the Harrison Narcotic Law by the Deputy Collector of Internal Revenue.

Dr. Edw. C. Kirk will deliver the principal address at the dedication of the Millem Memorial statute on Wednesday afternoon, followed by Drs. T. P. Hinman, T. W. Brophy, N. S. Hoff and others.

Thursday morning will be devoted to the presentation of a number of illustrated, descriptive clinics before the entire society and Friday forenoon to a large number of general table and chair clinics.

On Wednesday evening a banquet will be served for our guests and members.

It is the expectation that this meeting will set a new high mark in state society gatherings. Dr. Hinman, President and Dr. King, General Secretary, of the National Dental Association will be present and members from all state societies will be given a cordial welcome.

In view of the features of especial interest we hope to have representatives from every state, inasmuch as nearly every state contributed to the expense of the Miller Memorial.

Please note the four days session and meet with us if possible.

F. R. Chapman, *Secretary*,
305 Schultz Bldg., Columbus, Ohio.

### MEETINGS

IOWA STATE BOARD. Will meet for examination and registration at Iowa City, Iowa, December 6, 1915. Dr. J. A. West, Secretary, Utica Bldg., Des Moines.

TEXAS STATE BOARD. Will meet in Dallas, Dec. 13, 1915. Dr. C. M. McCauley, Secretary, Wilson Bldg., Dallas, Texas.

PENNSYLVANIA STATE BOARD. Will meet in Philadelphia and Pittsburg, December 15, 1915. Address for particulars, Department of Public Instruction, Harrisburg, Pa.

CALIFORNIA STATE BOARD. Meets in San Francisco, Dec. 9, 1915. Address for particulars, Dr. C. A. Herrick, 133 Geary St., San Francisco.

NEW JERSEY STATE BOARD. Meets in Trenton, N. J., Dec. 6, 1915. Address Dr. J. C. Forsyth, Secy., 430 E. State St., Trenton, N. J.

DISTRICT OF COLUMBIA EXAMINING BOARD. This Board will meet in Washington, D. C., January 3, 1916. Address Dr. Starr Parsons, Secy., 1309 L St., N. W. Washington, D. C.

MONTANA STATE BOARD. Will meet in Butte, Mont., January 10, 1916. Address Dr. C. A. Chevigny, Secy., Butte.

NORTH CAROLINA STATE BOARD. Will meet in Salisbury, January 13, 1916. Address Dr. F. L. Hunt, Secy., Asheville, N. C.

